# Extending care beyond the clinic: integrating patient-reported outcomes in chronic pain management through human factors engineering

**DOI:** 10.3389/frhs.2025.1474699

**Published:** 2025-04-23

**Authors:** Sadaf Kazi, Robin Littlejohn, Kelly M. Smith, Deanna-Nicole Busog, Joseph Blumenthal, Shrey Mathur, Zach McGill, Doug DeBold, Aaron Zachary Hettinger, Kristen E. Miller

**Affiliations:** ^1^ National Center for Human Factors in Healthcare, MedStar Health Research Institute, Columbia, MD, United States; ^2^ School of Medicine, Georgetown University, Washington, DC, United States; ^3^ Institute for Health Policy, Management, and Evaluation, Dalla Lana School of Public Health, University of Toronto, Toronto, ON, Canada; ^4^ Department of Patient-Oriented Research, Michael Garron Hospital, Toronto, ON, Canada; ^5^ Center for Biostatistics, Informatics, and Data Science, MedStar Health Research Institute, Columbia, MD, United States; ^6^ Vessel Partners, Minneapolis, MN, United States; ^7^ Center for Diagnostic Systems Safety, MedStar Health Institute for Quality and Safety, Columbia, MD, United States

**Keywords:** human factors engineering (HFE), chronic pain, opioid tapering, user-center design, patient-reported outcomes

## Abstract

**Objectives:**

Tapering prescription opioid pain medication through evidence-based guidelines can help in combating the opioid epidemic. Integrating clinical decision support (CDS) into the clinical workflow of tapering can help in translating guidelines to formulate and implement a tapering plan that manages pain symptoms while minimizing withdrawal, and optimally engages with the patient. The purpose of our project was to develop patient- and clinician-facing CDS in the area of chronic pain management in one integrated application (app) called Tapering And Patient Reporting outcomes for Chronic Pain Management (TAPR-CPM) App.

**Methods:**

We leveraged human factors methodologies and a user-centered design (UCD) approach through guideline review, stakeholder interviews, ethnographic workflow analysis, process mapping, design workshops, and usability testing. Participants included patients with chronic noncancer pain, their family members, pain management physicians, primary care physicians, and health IT developers who focus on patient- and provider-facing technologies.

**Results:**

Based on interview findings and workflow analysis, the provider-facing app had five sections: Patient Context, Taper Settings, Create Taper Plan, Withdrawal and Non-opioid Pain Plan, and Summary Dashboard. The patient-facing app had three sections: Maintaining a Pain Journal, Sharing Pain Scores with Provider, and Connecting to Resources about Opioid Tapering.

**Conclusions:**

This project leveraged a multi-method approach based in human factors and UCD to develop the TAPR-CPM app. Engaging with a diverse set of stakeholders including patients, caregivers, primary care providers, pain specialists, and health information technology developers was critical to develop a user-friendly experience with accessible technology to support patient engagement and provider decision-making.

## 1 Introduction

Chronic pain treatment and management requires innovative patient engagement and healthcare system strategies to inform decision making for both patients and clinicians. Chronic pain is a multidimensional health condition defined as pain persisting or recurring for more than three to six months (1). While the true prevalence of Americans living with chronic pain is difficult to define, as of 2021, an estimated 20.9% of US adults experienced chronic pain, translating to 51.6 million people and 6.9% (17.1 million) experienced high-impact chronic pain (i.e., pain that results in substantial restriction to daily activities) (2). Chronic pain complaints are the second most common reason for outpatient primary care visits (3). Pharmacological management of pain—including opioid analgesics—is often a first line of defense for many clinicians (4). Despite inadequate evidence of long-term benefit, 3%–4% of US adults report long-term use of opioid medications (5). Given the prevalence of opioid prescriptions more broadly, the treatment and clinical management of chronic pain is among the most vexing challenges currently facing primary care providers (PCPs) (6).

Prescription opioid pain medication overuse, misuse, and abuse have been significant contributing factors in the opioid epidemic. Healthcare systems are moving towards optimizing pain therapy through opioid-dose reductions, (i.e., opioid tapering). However, implementing opioid tapering is exacerbated by sociotechnical challenges including a limited number of pain specialist physicians and patient pessimism about non-opioid treatments for pain and fear of opioid withdrawal (7, 8). Although PCPs provide much of the healthcare systems chronic pain management, they report a number of challenges: minimal training in pain treatment and management, a lack of resources to support opioid tapering decisions, practical time constraints to address optimization of pain therapy in a routine visit, and maintaining the provider-patient relationship through challenging communications characterized by highlighting the importance of tapering and managing patient fears of being abandoned by providers during the taper (9–12). The science of human factors engineering and user-centered design can help address these unique challenges faced by providers and patients to design user-friendly solutions to support opioid tapering for chronic pain management.

One potential solution is the use of clinical decision support (CDS) to enhance health-related decisions, action, and outcomes. CDS strategies enabled by modern health information technology (health IT) offer more targeted opportunities to provide information when, where, and how it is needed to optimize patient and care decisions, actions, and partnerships. CDS also provides the opportunity to capture patient perceptions about outcomes meaningful to them such as level of functioning with pain, quality of life, and satisfaction with the care team and treatment, i.e., patient-reported outcomes (PROs) (13–16). Given the dangers of opioid medications as first-line treatment for chronic pain, the need for such measures is especially imperative. Consequently, national guidelines and experts have called for the assessment of pain-related functioning in addition to pain intensity to determine whether patients are benefitting sufficiently to merit the use of opioid treatment or whether lower doses of medication and/or nonpharmacological treatment options should be prioritized (17–19). Despite the recognition of the potential benefits of using functional pain-related PROs, their systematic use in everyday clinical care is rare.

Designing a CDS system tailored for patients undergoing opioid tapering for chronic pain necessitates a rigorous human factors engineering approach. This methodology is crucial as it emphasizes the integration of human capabilities, limitations, and preferences into the system's design and development. By focusing on human factors science encompassing the concepts of cognitive processes, usability, and user-centered design principles, the CDS can effectively support patients in navigating the complex and often challenging opioid tapering process (20). Understanding user needs and behaviors ensures that the system enhances patient engagement, promotes adherence to tapering protocols, and ultimately improves clinical outcomes while minimizing the risk of opioid misuse or relapse (21, 22). Thus, applying human factors engineering to the design is essential for creating a supportive, intuitive, and safe tool that optimally serves patients.

The objective of this paper is to describe the user-centered design (UCD) approach involved in developing and informing the implementation of a CDS system for chronic pain management with two components: patient-facing CDS and clinician-facing CDS in one integrated application (app) called: Tapering And Patient Reporting outcomes for Chronic Pain Management (TAPR-CPM) App. Our approach tackled technological and design components of health IT architecture while understanding end-user needs (e.g., patients and clinicians), workflow, and data integration. We describe a “codesign” approach whereby we engaged true end users in the development of the TAPR-CPM app. This approach is grounded in the concept of co-production (23–25) informed by empirically validated models supporting patient and clinician behavior change (26) and effective approaches for translating evidence into practice.

## 2 Methods

### 2.1 Study population and design

We leveraged human factors methodologies and a UCD approach through guideline review, stakeholder interviews, ethnographic workflow analysis, process mapping, design workshops, and usability testing. Stakeholder feedback was elicited at several stages throughout the knowledge discovery and pre-implementation design phases of app development to capture the needs of the intended end users, i.e., patients with chronic pain, pain management physicians, and PCPs. The study was approved by the MedStar Health Research Institute Institutional Review Board.

Data collection efforts purposefully sampled a heterogenous sample of participants. Participants included patients with chronic noncancer pain, their family members, pain management physicians, primary care physicians, and health IT developers who focus on patient- and provider-facing technologies. Some participants contributed to a single activity; others participated in multiple activities.

### 2.2 Data collection instruments and procedures

#### 2.2.1 Guideline review

We reviewed guidelines and best practices on tapering opioids for chronic noncancer pain to inform the CDS. The search strategy for guidelines relevant on opioid tapering was decided in conjunction with clinical subject matter experts (SME), including pain management specialists, psychiatrists, and primary care physicians. Federal guidelines (e.g., from the Veterans Affairs/U.S. Department of Defense, Centers for Disease Control and Prevention, U.S. Department of Health and Human Services) and peer-reviewed literature on opioid tapering were reviewed by physicians for alignment with knowledge and practices about tapering, guidance specificity and clarity, and gaps and discrepancies between the guidelines (27–31). Concurrently, we conducted a task analysis to organize the key tasks performed by physicians during the process of opioid tapering, and analyzed which key tasks and decisions were supported by the guidelines. We validated the task analysis with clinical SMEs, including pain management specialists, psychiatrists, and primary care physicians.

#### 2.2.2 Semi-Structured interview

**Figure 1:**
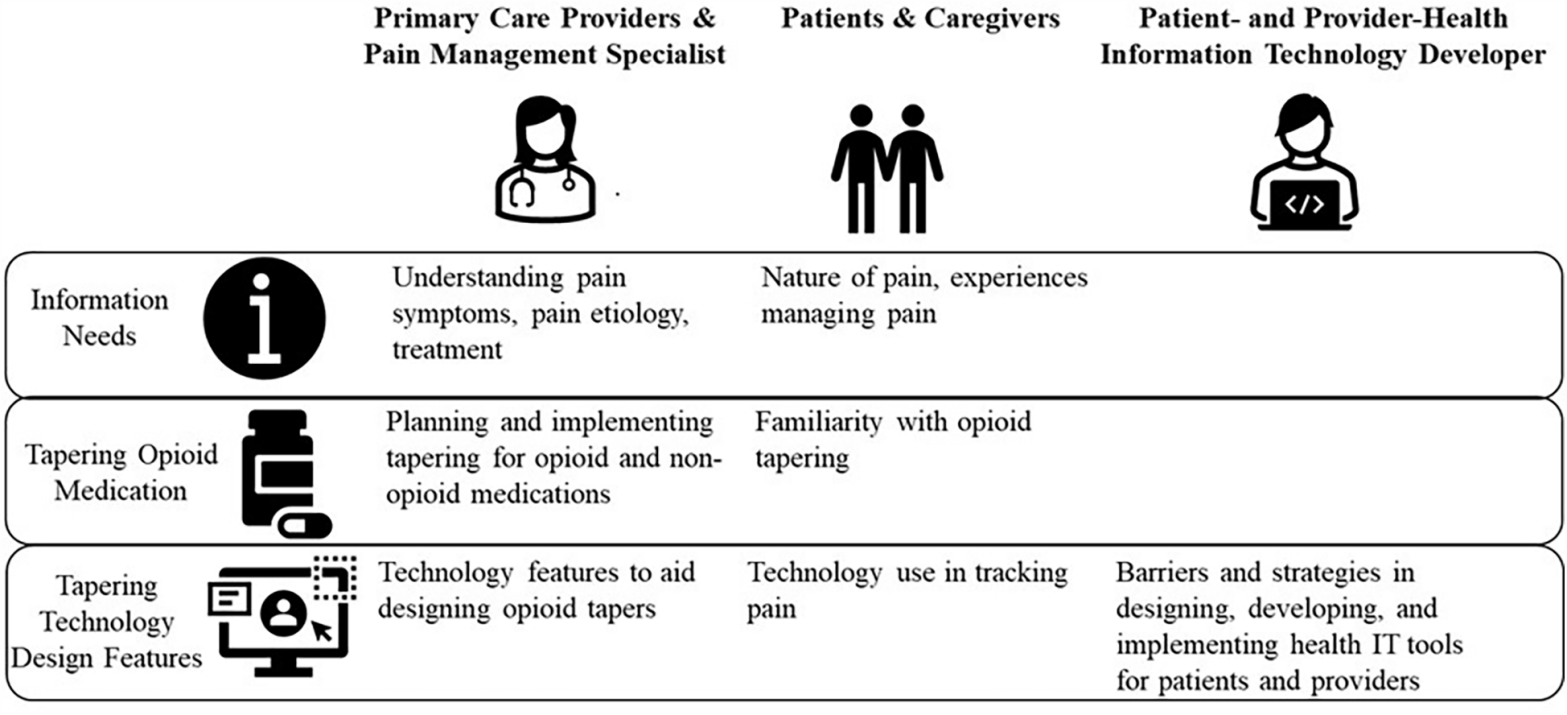
Interview topics for healthcare providers, patients, caregivers, and patient- and provider-facing health information technology developers.

We developed five interview guides, one each for patients with chronic pain and their caregivers, PCPs, pain specialist providers, patient-facing health IT developers, and provider-facing health IT developers. Figure 1 lists the interview topics for each participant group. Remote interviews, lasting no more than an hour, were conducted by interviewers skilled in human factors or implementation science. Interviews were audio recorded, de-identified, and transcribed for analysis.

#### 2.2.3 Workflow analysis

The goal of the workflow analysis was to understand how the CDS tools will function under realistic care setting conditions (e.g., variable workflows, high stress tasks, frequent missing data, interruptive environments) and the effectiveness and usefulness of tapering guideline recommendations. The workflow for tapering opioid medication was constructed by collating findings from interviews, guideline review, and electronic health record (EHR) data (e.g., details around the specific medication type, dose, and frequency). Workflow maps detailed cognitive tasks involved in implementing opioid guidelines [e.g., calculating the desired oral morphine milligram equivalent (MME)] for each tapering period and visualizations to guide prescribing patterns and patient-provider communication. The workflow maps were presented to SMEs for input to inform app design for usability testing.

#### 2.2.4 Design workshops and usability testing

Interview findings and workflow analysis were used to inform wireframes (i.e., two-dimensional illustrations of an app interface) for the patient- and provider-facing app through design workshops with a multidisciplinary team comprising clinical SMEs, human factors engineers, and informaticians. Participants brainstormed the content, design of app sections, and specific items for both apps. Prototypes were revised and finalized through several rounds of design sessions and formally evaluated through two rounds of usability testing. In Round 1, usability testing participants were allowed to freely explore the interface; iterative changes were made to address points of difficulty or confusion. Round 2 usability testing asked participants to perform specific tasks. Final changes to the interfaces addressed outstanding issues and points of confusion. A trained usability specialist conducted each session after completing a full verbal consent process with the participant. The usability specialist was able to give the participant navigation abilities, allowing them the ability to click through the app prototype. For the provider-facing app, use cases were designed with simulated patient data to simulate differing complexity levels in terms of opioid medication type (long- vs. short-acting opioids), patient history, and desired tapering speed (standard, slower than usual, faster than usual). For the patient-facing app, patients were asked to explore all components of the app but were not asked to enter their personal data into the app. For both provider and patient usability testing, sessions were one-on-one, conducted remotely, and lasted for approximately an hour.

### 2.3 Analysis

Sociotechnical systems theory was applied to summarize findings from all primary (i.e., interviews, usability testing) and secondary (guidelines review, workflow analysis) methods to understand systems barriers and facilitators in addition to specific design components. A sociotechnical system's perspective provides insights into external and internal organization systems influences (e.g., social, technical, environmental factors). Raw data from primary data collection methods (interview transcripts and usability sessions) were analyzed using the grounded theory approach to enable prioritizing participant narratives in guiding key findings to shape app design (32). Data were analyzed by identifying common themes across patient and provider groups. Focused coding was used to organize and synthesize the initial data. Once the data were coded, analysis was completed to determine the most prominent themes in the context of the interview questions and goals of the questions.

## 3 Results

Table 1 shows the details about participants at different stages of the design.

**Table 1:** Demographics of stakeholders across five activities including guideline review, interviews, design workshops, usability testing, and workflow analysis.

Method	Stakeholder type	*n*	Demographics
Guideline Review	Healthcare providers	6	Medical specialties: Pain management, palliative care, primary care
Interviews	Healthcare providers (Primary care)	4	Gender: Female (*n* = 4) Education: MD (*n* = 3); PharmD (*n* = 1)
	Healthcare providers (Pain management)	4	Gender: Male (*n* = 4) Education: MD (*n* = 4) Experience: 13–21 years
	Patients with chronic noncancer pain	4	Gender: Male (*n* = 3); Female (*n* = 1) Age: 58–76 years old; mean 68.1 Race: Caucasian (*n* = 3); African American (*n* = 1) Education: High school to advanced degree
	Caregivers of patients	4	Gender: Female (*n* = 4) Age: 50–76 years, mean 66.5 years Race: Caucasian (*n* = 3); African American (*n* = 1) Education: High school to advanced degree
	Patient-facing health IT developers	4	Experience: 2–6 years health IT experience
	Provider-facing health IT developers	4	Experience: 3–10 years health IT experience
Design Workshops	Research team (clinical and non-clinical members)	N/A	Specialties: Human factors, emergency medicine, health IT developers, implementation scientists, nursing, patient advocates
Patient Usability Testing	Patients with chronic pain	5	Gender: Female (*n* = 5) Age: 45–64 years Race: Caucasian (*n* = 4); African American (*n* = 1) Education: High school to advanced degree
Provider Usability Testing	Healthcare providers	10	Education: MD (*n* = 9); PharmD (*n* = 1) Experience: 4–16 years, mean = 10.4 years Specialties: Pharmacist (*n* = 1), Primary care (*n* = 8), pain management (*n* = 1)
Workflow Analysis	Research team (clinical and non-clinical members)	N/A	Specialties: Human factors, emergency medicine, pain management, health IT developers, implementation scientists, nursing, patient advocates

### 3.1 Provider-facing app

Based on guideline review and the workflow analysis, we identified three main tapering tasks for providers: identifying candidates appropriate for opioid tapering, implementing the tapering plan, and monitoring the safety of tapering. Provider interviews and input from SMEs showed that identifying tapering candidates was not challenging compared to the latter two tasks.

PCP 1: “*Let me put it to you this way. I am aware of the CDC mme (morphine milligram equivalent). I have this (patient I was) telling you about that I'm (tapering) in the next few weeks (who) is going to be unpleasant (and is on) about three times that (of the CDC mme). There's other people that are nowhere near that number, and they need to be tapered. So I would say, no (I don't think there is a specific number of mmes that is a trigger for opioid tapering)*”.

Further, PCPs had low familiarity with information to plan a taper, detailed steps outlined in opioid tapering guidelines about recommended speeds of tapering, dosage reductions, and implementing supportive therapies to manage withdrawal and pain.

Interviewer: *“So generally, if a patient tells you, “I'm ready to taper my opioids”, what do you generally do with that patient?”*

PCP 3: *“I don't think a lot of people know exactly how to taper … what percentage to go down by over what amount of time. I think that even more people have no idea of what withdrawal medicines to use. So … because now I've read the (CDC) guidelines that you sent me, I'm like, “Oh, well, now I know what to do”. But … before I read that, depending on if they were only on short acting (medication) like Oxycodone … say they were taking like, five of Oxy four times a day. I might see if I could … get a couple of those to be half a pill, or, you know, be like, “Okay … take it three times a day, and then at night, just take half a pill”.*

On the other hand, pain specialist providers reported extensive experience setting boundaries and expectations with patients prior to beginning opioid therapy and in implementing and monitoring the impact of opioid tapering on patients’ physical functioning (e.g., pain intensity and interference with activities of daily living) and mental health (e.g., depression) through PRO measures. Based on information needs elicited from interviews and the workflow analysis, we decided to focus the provider-facing app to support PCPs in formulating and executing a tapering plan and monitoring its impact on patients.

**Figure 2:**
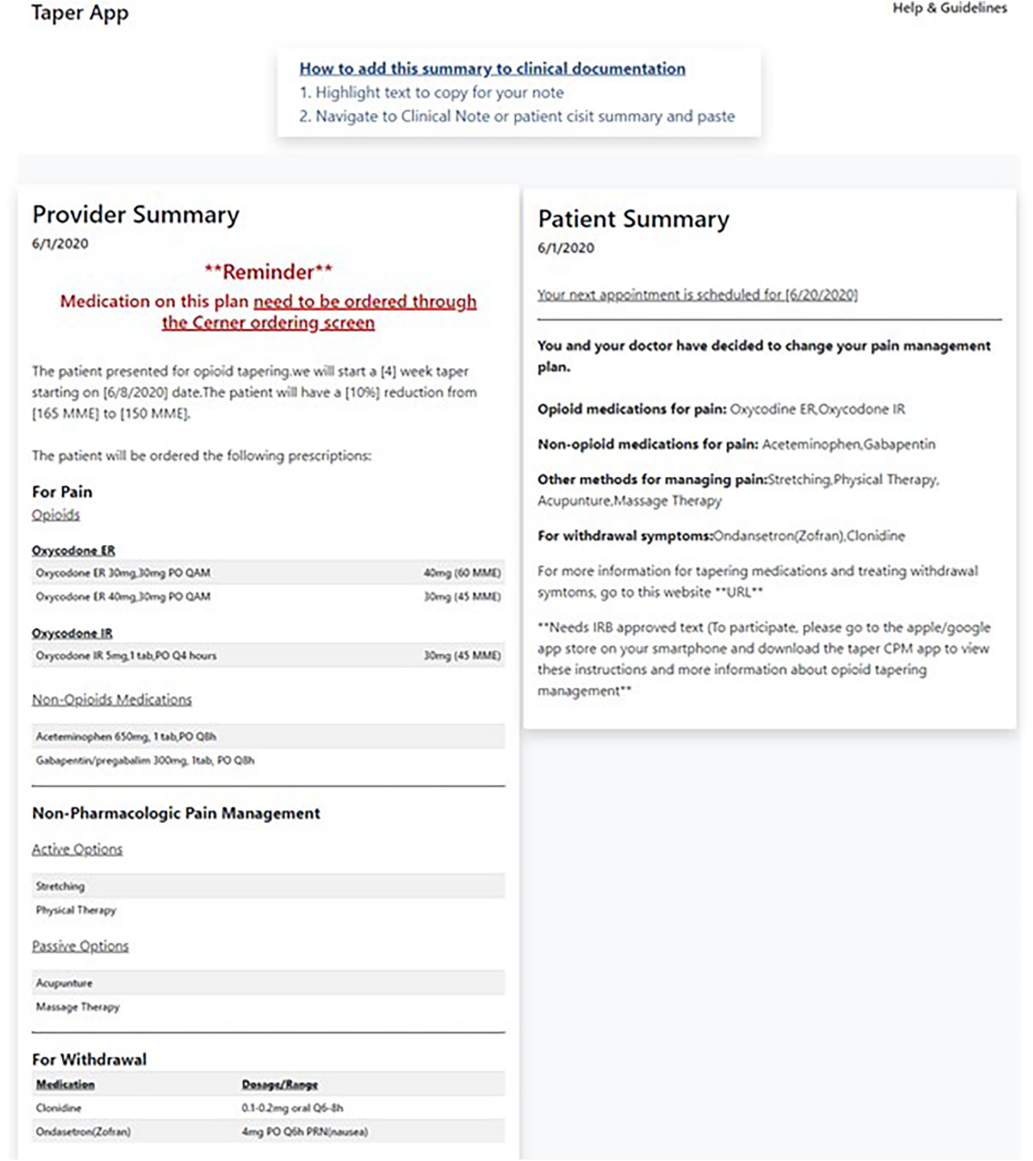
The TAPR-CPM provider summary allows providers a final check of the tapering plan and associated medications after using the app and provides flexibility for the providers to incorporate the findings into the EHR as it aligns with their workflow.

Through design workshops and usability testing, we prioritized three main goals for the provider-facing app: (1) operationalize technical guidelines for prescribing and tapering opioids for chronic pain to address information gaps about tapering speed and dose, (2) better monitor functional pain and opioid use through PROs that include depression measures and incorporate a range of alternative strategies for pain management, and (3) visualize patient data. We synthesized these findings to create five sections in the provider-facing app: Patient Context, Taper Settings, Create Taper Plan, Withdrawal and Non-opioid Pain Plan, and Summary Dashboard. The Provider Summary Dashboard is created after interaction with the first four sections of the TAPR-CPM app (Figure 2). The sections of the TAPR-CPM app are discussed below.

#### 3.1.1 Patient context

Interviews with PCPs revealed a need for integrated information pertinent to understanding the patient's relevant opioid medication management history.

PCP 2: “*…The app would enable reviewing the prescription drug monitoring program (PDMP) there as opposed to having clicked in and clicking out. Maybe it could also connect to the patient's pain contract and also the most recent drug screen? So that we have one tab to go to to review everything to meet our requirements. So, we've reviewed the PDMP. We reviewed the last pain contract. “Oh, look. It's been over a year. We should probably redo that today”. And “Oh, they haven't had a random urine screen in six months. We're going to do that today as well*”.

PCP 4: *“A dedicated section for, When was this person last referred to physical therapy or orthopedic?” Maybe the date… Put those pain management notes in one section. And then maybe, their musculoskeletal MRIs or CTs in one place, because then you could quickly see, “Okay, when's the last time we did look at this person's neck or low back, or what have you?”*

Based on these findings, we designed the Patient Context section to provide an overview of pertinent patient clinical data summary including, laboratory testing, medication lists and integration of the PDMP.

#### 3.1.2 Opioid tapering medication plan

Interviews with PCPs showed they desired support with creating a tapering schedule based on morphine milligram equivalents of all the medications that patient was on.

PCP 2: “*Maybe, (what will) be helpful is what they're currently on in, like morphine equivalents. That would be easy if it automatically calculated what's in their med list and what their daily … morphine equivalents are. And then … you could put in what their decreased dose was and see how much they're decreasing every day. That would be nice. Or even to convert between one medicine to another one. So, if you're going to be going from a long acting (opioid medication) to a short acting (opioid medication), to help convert your (morphine milligram) equivalent there. That would be very helpful*”*.*

In addition, providers also asked for help in generating a tapering schedule that providers could implement.

PCP 1: “*I think it would be great if it generated an actual (tapering) schedule. So somehow you put in what the patient's on, and hit a button, and this thing generates a schedule*”.

Based on these findings, the Opioid Tapering Medication Plan section is designed to support choosing the tapering plan in a stepwise fashion. It presents providers with the patient's current list of opioids and aids in selecting the initial tapering plan and calculating the oral MMEs. It also broadly presents options for tapering speeds (standard, slower than usual, and faster than usual tapers) as a starting point. Our workflow analysis found that MME calculation was a cognitively complex task with high potential for miscalculation error; therefore, we decided to automate MME calculation.

#### 3.1.3 Create taper plan

This section of the app enables specifying details about the tapering plan selected in the previous screen. Providers wanted the option to manually input varying tapering speeds and compare tapering plans with different speeds.

PCP 3: “*I'm just imagining it in my head, if there was almost an option to change the percent to go down by per month or week, so that I could see what the different … sort of see what that (i.e., different plans) would look like*”.

Our workflow analysis revealed that dose modification involved multiple steps which could result in high cognitive workload and potential for error if providers had to perform these calculations in conjunction with choosing the tapering speed. Therefore, to avoid overwhelming providers with multiple decision points on a single screen, we chose to separate these two tasks. The app first asks providers to choose the tapering speed. Then, the Create Taper Plan section enables providers to modify specific details about the opioid medications (e.g., long- vs. short-acting, dose, frequency) to reach the target oral MME dose for the upcoming taper period corresponding to the desired tapering speed chosen on the previous section.

#### 3.1.4 Withdrawal and non-opioid pain plan

Many providers mentioned proactively managing expectations about pain and treating withdrawal symptoms with a goal of minimizing the impact of experiencing withdrawal on patients' lives:

PCP 1: *“So, I usually say at the outset, you know, “We're gonna learn to manage your pain. We're not gonna make it away. There's no manage magic bullet here. It's all about function.” Is that (pain) also related to withdrawal symptoms? And … that's a big part of this game, is having people know about withdrawal. Withdrawal is incredibly … painful, and it's just … so uncomfortable. And so, a lot of people, they just live in fear of withdrawing. Which I understand, if they're taking their meds, right? They can go to work, they can take care of their kids, yeah, but if they go and start having all that stuff (experiencing withdrawal symptoms), they're out, they can't live their lives”.*

PCP 3: “*…Having … a good outline of those (withdrawal medications) is really helpful*”.

Provider and patient interviews helped us generate many sources of non-opioid and non-pharmaceutical therapies to manage pain (e.g., physical activities such as yoga, stretching, physical therapy; non-traditional treatments such as acupuncture, massage therapies).

*PCP 1:* “*I absolutely suggest yoga. I suggest weight loss programs, if I think that's part of it*”*.*

Pain Specialist 3: “*Some combination of medications, physical therapy, psychological therapies … I'll recommend alternatives sometimes, like acupuncture, chiropractic, different modalities, injections*”.

Guideline review and SME inputs helped identify non-opioid therapies to manage pain (e.g., non-steroidal anti-inflammatory drugs) and monitor their appropriateness through relevant lab results (e.g., liver and/or kidney dysfunction), and medication to manage withdrawal symptoms from long-term opioid therapy.

PCP 3: “*I tend to lean pretty heavily on physical therapy, NSAIDs and ice and heat and stretching and all that stuff*”.

Based on these findings, the Withdrawal and Non-Opioid Pain Plan section serves as a checklist of options to proactively treat withdrawal and integrate the patient's medication list and laboratory testing that may impact the selection of certain medications. Our goal was to facilitate clinician decision making and prevent the clinician from having an error of omission by forgetting to treat the patient's pain or waiting for the patient to go into withdrawal before prescribing appropriate medications. All providers appreciated the holistic approach to opioid tapering that concurrently addressed withdrawal symptoms and ongoing pain experienced during opioid tapering.

#### 3.1.5 Summary dashboard

This section ensures that clinicians can conduct a final review, catch any potential errors, discuss and share the opioid tapering plan with the patient before finalizing it, and integrate reports into the EHR.

#### 3.1.6 PRO data

Provider interviews revealed the importance of evaluating the impact of pain medication on pain levels and the patient's functioning.

PCP 3: “*I usually ask them, “How's your pain been since I last saw you? Would you say that it's worse, better, about the same?” But I usually ask them then to describe the pain again to me, and depending on the type of pain, like things like headaches, I'll usually help them quantify, like, “How many days a week did you have the pain? How long did it last for?” And then ask them if they've identified any, triggers or things that have helped*”.

We used to these findings to design the PRO Data section. After the initial visit, the PRO section provides a visualization of PROs following the initial tapering period and app use, patient journal data to provide additional context around the patient's experience, and a dedicated screen for medication plan to add structure for creating the next taper interval. As a result of patient interaction with the patient-facing app, subsequent clinical encounters could leverage this data to inform decisions to optimize the tapering process.

### 3.2 Patient-facing app

Based on interviews, we identified that patients and their caregivers perform substantial work to track details about their pain and to communicate that information to providers.

Patient 3: “*We use the calendar to track the day, the pain level, what meds we take, and our activity*”.

Patients also mentioned the mental toll of tapering in terms of its impact of their functioning.

Patient 2: “*After first starting that lower dose that I felt horrible. I missed a lot of work, actually. So it has a huge impact on your ability to function … because my body was so used to it. And whatever that part of the brain is that it's the feeling of the drugs was saying, “Hey, you're not giving me what I really want.” And it took a lot of mentally fighting that off to say, “No, what I really want is to go down (reduce opioid dosage)*”.

All patients actively engaged with their providers to discuss pain, set goals, and current treatment options through a variety of methods (e.g., phone, patient portal, email).

Patient 3: “*If it hurt, I took something. If it didn’t hurt, I didn’t take it. You know, it wasn’t like I have to have this (substance such as alcohol, nicotine) to function, and never got into that. Still haven’t and my doctor and I have worked pretty hard over the years to make sure that [further increase of opioid dosage] didn’t happen*”

Patients prioritized clear communication across multiple members of their care team (e.g., primary care physician, pain management specialist).

Patient 1: “*If I experience paralyzing pain, I call the doctor, and he will explain things to me about the pains and everything*”.

**Figure 3:**
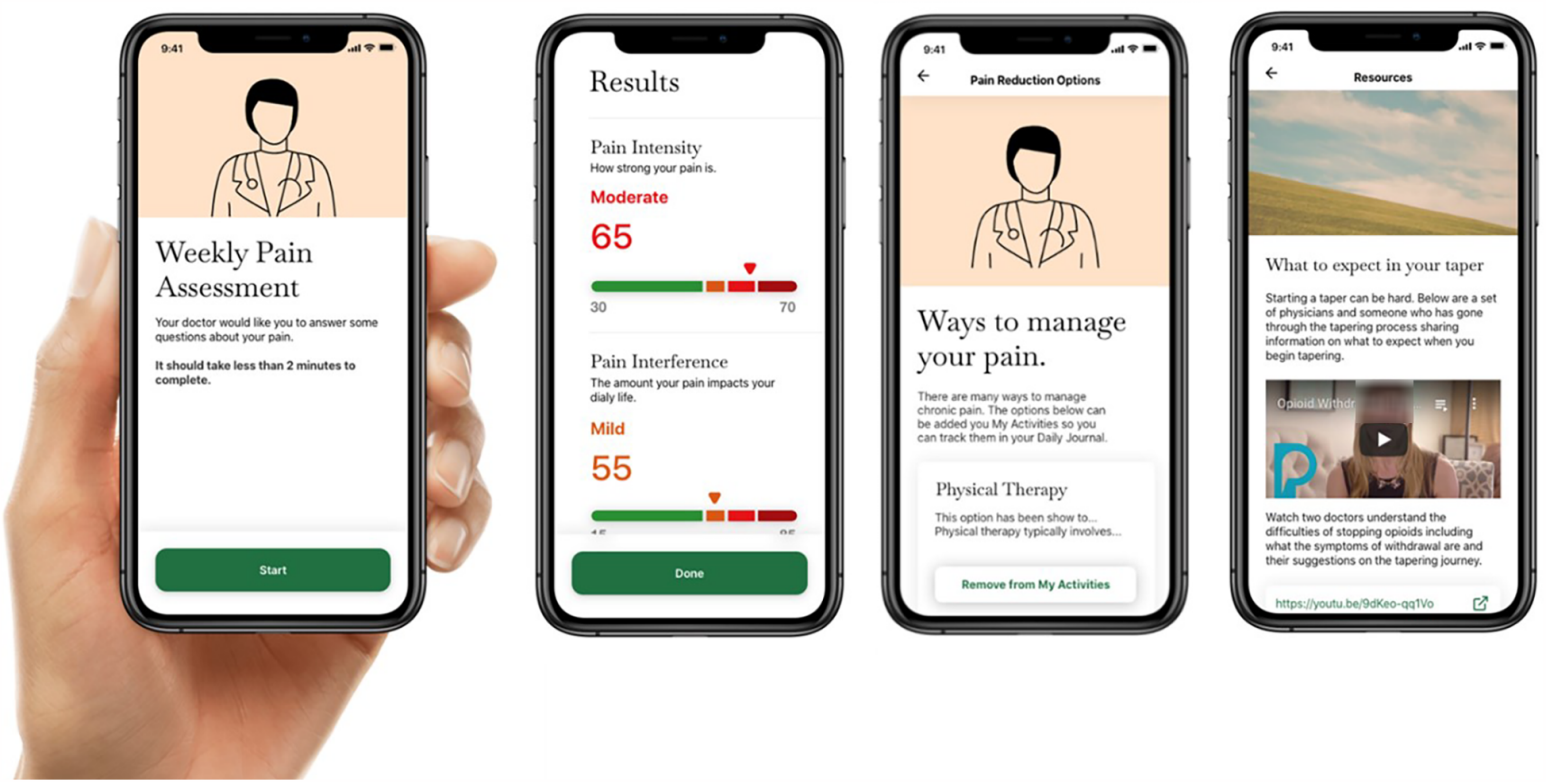
Screenshots of the patient-facing TAPR-CPM app illustrate different components including weekly pain assessments, taper plan monitoring and progress, and education resources.

Communication was particularly important during an active taper to manage the patient's experiences and unexpected physical and mental effects of the taper. Therefore, through design workshops, we prioritized three main goals for the patient-facing app: (1) tracking information about pain symptoms, (2) sharing relevant information with providers, and (3) getting connected to resources to better understand the tapering journey. We synthesized these findings to create three sections in the patient-facing app: Maintaining a Pain Journal, Sharing Pain Scores with Provider, and Connecting to Resources about Opioid Tapering (see Figure 3).

#### 3.2.1 Maintaining a pain journal

Many patients highlighted how pain levels vary over time, sometimes in response to chronic conditions, emphasizing the value of a journal to track daily changes and identify patterns over time.

Patient 2: “*Something in the app to track pain daily where it would ask: “What's your pain level now? What have you been doing?” So that you can see that my pain level has grown by doing these certain things, my pain level has decreased by doing other certain things*”.

Patient 3: “*I got Lyme disease and everything changed. We’ve been dealing with that nerve pain ever since. It's really trying to get back to that “before” state, or somewhere closer to that. You never expect to go 100% back but you’d like to be able to do some of the things you did*”.

Provider interviews also revealed potential positive impacts of pain tracking on patients.

Pain Specialist 3: *“There's probably a lot of potential there … to allow the patient to be tracking their progress also. So, seeing a trend line for their morphine equivalents and their pain numbers, especially if they're both going in a good direction, could be really interesting … some kind of … patient reassurances”.*

Based on these findings, we designed the “Maintaining a Pain Journal” section to enable patients to track their daily record of pain localization on a visualization of the body map tool, and understanding the pattern of their pain symptoms over time, and track daily mood through emoji sets, as well as daily activities.

#### 3.2.2 Sharing pain scores with provider

Patients discussed the importance of sharing contextual factors influencing medication usage or pain levels.

Patient 2: “*If one of the (transdermal) patches had actually fallen off … it just would let them know that's why there was an uptick in the oxycodone*”.

Therefore, we designed the Sharing Pain Scores with Provider section to enable patients to record their pain intensity and pain interference scores on a pre-set day each week (e.g., Monday), share their pain scores and symptoms with their physician, and review their scores with the physician at their next visit.

#### 3.2.3 Connecting to resources about opioid tapering

Providers and patients revealed several resources that could help patients during the taper process, including anticipating withdrawal symptoms, and information to help with tapering doses.

Patient 2: *“Seeing a psychologist or someone like that…adding that person in to be able to talk to and have them give you alternate suggestions (on how to manage pain without opioids). A discussion board where you could post to your success, like, “Hey, this worked for me.” Or, “I had this side effect.”*

PCP 3: *“I think patients tend to do better if … they're fully aware of what to expect. And so, I can go through like, “Oh, these are the symptoms of withdrawal”. But, you know, if I had a way to be more specific … usually, like, “X many hours from your last dose is when you're going to start feeling bad. Here's usually how it starts … things will probably be worse at this point, and then things should start to get better”. If a patient has … a very clear understanding of the trajectory, like, “Oh, 48 hours is when I'm going to feel the worst. Things should be turning around. I can power through a little bit.”*

Patient 3: “*For me, because I like keeping track of when I can do things just so I can have my own information. Like the fact that when you're sick like this, when you have chronic pain, looking back at what you can do on certain times is like a reminder. Like “Wait a minute, I've experienced this before. When was that?” Go back through the notes. “Okay, this is what I was able to do that time. Let me try and do that”. Because you forget that sometimes you're able to push through it and do certain things*”.

Therefore the Connecting to Resources about Opioid Tapering section was designed to provide resources to support patients throughout the tapering journey. These resources include understanding what to expect during an opioid taper in terms of dose reductions and its effect on pain and other symptoms, ways of managing pain and other symptoms during a taper, experiences of other people with opioid tapering, and social support resources that can be accessed to aid during the tapering journey.

### 3.3 Workflow analysis

Patient and provider workflows for tapering opioids in primary care settings were created for four high-level tasks: pre-taper; initial visit; home experience, and follow-up visit. Table 2 shows sub-tasks under each higher-level task. The workflow analyses identified several gaps between the ideal vs. actual workflows (i.e., work as imagined vs. work as performed). Providers must often go to different places on the EHR to assess the patient's candidacy for taper, gather information to plan the taper, and review effectiveness of the taper. Several findings from provider interviews re-surfaced in workflow assessments: there tends to be a lack of support for operationalizing evidence-based guidelines at the point of care, resulting in sub-optimal taper plans, which fail to consider recommended taper parameters and holistic management of withdrawal and pain symptoms. There is also a relative lack of standardized patient-friendly resources to engage patients during opioid tapers and educate patients about what to expect during opioid tapers. We designed our apps to address many of these challenges.

**Table 2:** Overall tapering workflow organized by activities of the patient, provider, TAPR-CPM app, and EHR workflow across the four stages of pre-taper, initial taper visit, activities that occur outside of the clinic visit (“home”), and follow-up visit.

		Taper setting			
		Pre-taper	Initial taper visit	Home	Follow-up visit
Stakeholder workflow	Patient	• Self-assess willingness to taper • Self-educate (opioids, tapering) • Educate provider on history (medical, social) • Commit to shared decision making with the provider	• Update history (medical, social) with provider • Participate in plan development (opioid tapering, withdrawal symptoms management, non-opioid pain management) • Commit to open communication regarding taper success/challenges • Reconfirm commitment to shared decision making with provider	• Explore the app (features, resources, data) • Review prescribed plan development (opioid tapering, withdrawal symptoms management, non-opioid pain management) • Continue commitment to open communication regarding taper success/challenges	• Update history (medical, social) with provider • Participant in plan modification (opioid tapering, withdrawal symptoms management, non-opioid pain management) • Continue commitment to open communication regarding taper success/challenges • Continue commitment to shared decision making with provider
	Provider	• Assess taper candidacy • Educate patient (opioids, tapering) • Ensure open dialogue with patients about options for tapering	• Review patient history • Educate patient [e.g., through patient-reported outcome measurement information system (PROMIS), risks] • Self-educate (tapering guidelines) Obtain patients opioid history • Create/prescribe/document plans (opioid tapering, withdrawal symptoms management, non-opioid pain management) • Reconfirm commitment to patient/provider relationship		• Review patients updated history • Review patients data (from app and patients verbal updates) • Update/prescribe/document plans (opioid tapering, withdrawal symptoms management, non-opioid pain management) • Continue commitment to patient/provider relationship
	Tool		• Provide education and resource links • Calculate taper • Document and save entered information • Generate summary documentation • Generate data visualizations	• Provide education and resource links • Calculate taper • Document and save entered information • Generate summary documentation • Generate data visualizations	• Provide education and resource links • Calculate taper • Document and save entered information • Generate summary documentation • Generate data visualizations
	EHR	• Clinical documents, medication, and Prescription Drug Monitoring Program [Chesapeake Regional Information System for Patients, or CRISP] data	• Clinical documents, medication, and CRISP data		• Clinical documents, medication, and CRISP data

### 3.4 Technical specifications

During the course of the design and implementation of the apps, several key design decisions were made based on our user-centered design approach. We included patient portal authentication and designed a “lite” version of the app to test pain tracking and optimize EHR workflow. The “lite” version solution was designed for sites that could not accommodate an embedded FHIR app within their EHR. This version used a “hubless” application model (lack of data hub) that was connected to the EHR and easily accessible without requiring the provider to leave their current workflow. The elimination of the data hub requirement reduced the technical requirements of the application and expedited implementation.

#### 3.4.1 Authentication of patient portal

Based on feedback from providers and health IT developers, the research team decided early in the design process to leverage the health system's patient portal for authentication and secure data transfer. The use of the patient portal allows the patient to use a single set of credentials and reduce maintenance of a redundant authentication strategy. Patient portal authentication does add complexity for the patient if they do not already have a portal account or do not have their login credentials easily accessible.

#### 3.4.2 “Lite” app version for chronic pain tracking

A second design decision led to a streamlined “lite” version of the app created to focus on pain tracking. The initial implementation of the patient and provider app provided two-way communication between the provider and patient, and the creation of opioid tapering plans. Clinicians at later stages in the project requested streamlined approaches to help track patients’ pain experience before opioid tapering was initiated as well as a focus on tapering calculations before providing two-way communication.

#### 3.4.3 Optimizing for EHR workflow

Different EHRs provide clinicians the ability to document via different pathways and in different locations. Guided by best practices in human factors engineering, we decided that the content created by the provider app should be optimized to the individual EHR to make sure it matches the provider's workflow in terms of size of text recommendations and formatting. For example, copying and pasting large amounts of text into small text entry boxes may reduce the opportunity for the clinician to make modifications of the plan that is placed in the patient's chart.

#### 3.4.4 Design for safety

The clinical team provided input on the medications and dosing that would be appropriate for tapering opioids. For example, the app was limited to medications where conversion factors were readily available with a maximum number of opioids set to two (including only one long-acting opioid). Safety guardrails prevented tapering in patients with more complex medical histories or patients that may have duplicate or out of date prescriptions in their record. Implementation teams may decide to expand or reduce the number and types of opioids allowed for tapering based on local prescribing practices.

## 4 Discussion

A human factors engineering, user-centered design approach elicits feedback from stakeholders and provides an opportunity to collaborate and co-design with representative end users as sessions support creative thinking and the generation of ideas and solutions (33). This research identified design features for the TAPR-CPM app and facilitators and barriers for implementation of CDS to streamline the delivery of care. The design of the provider-facing app supported organization of EHR data that is likely to reduce the need to “hunt and gather” (i.e., identifying and reviewing multiple individual tabs including medications, patient history, and diagnoses). Designing a solution that incorporates individual components into a single app can allow providers to assess candidacy, plan a taper, and assess the efficacy of a taper. Providing support to operationalize tapering guidelines at the point of care, a need elicited through PCP interviews, can encourage evidence-based medicine by supporting the workflow of modifying the taper plan. The design of the patient-facing app can support shared decision making through patient-provider feedback (e.g., reporting taper effects, resources related to chronic pain) and encourage patient engagement by providing educational resources and an education plan (e.g., resources related to tapering, what to expect, when to contact your provider). Collectively, the design promises to facilitate an effective patient-provider partnership during the opioid taper. Lastly, TAPR-CPM app facilitates a holistic approach to tapering that includes integration of standard patient-reported outcome measures for patients to report pain symptoms between appointments.

Successful adoption of CDS requires careful consideration of the knowledge driving the alert system (technical integration) and also requires application of human factors principles to understand the system (20, 34). There is substantial value in clinical, operational, and technical understanding and validation of newly developed CDS in the stages that occur prior to public release. Stakeholder interviews ensured input from end users and perspective of developers experienced in creating patient- and clinician-facing technologies was incorporated into the ultimate design. A comprehensive evaluation of current state guideline implementation with considerations of EHR use, clinical decision-making, and shared decision-making was used to support development of the implementation strategy. Workflow analysis was used to evaluate the current state of primary care clinician-patient interaction using an ethnographic approach to develop site-specific process maps that identified the overall processes and specific elements for CDS implementation. Usability testing included a formative evaluation of preference and performance to assess usability before going live and to allow time for iterative changes. The application of these methods ensured inclusion of stakeholder feedback, considerations of workflow, and review of adherence to design principles.

Specifically, many existing CDS tools for chronic pain management focus exclusively on either the clinician or patient side of the equation (35). TAPR-CPM is distinct in its integration of both patient-facing and clinician-facing components into one application, allowing for a more cohesive approach to managing chronic pain. Unlike other initiatives that focus primarily on static educational content or decision aids for clinicians, TAPR-CPM incorporates dynamic features informed by co-design with end users. This approach has been seen in recent studies co-design pain management programs with patients, underscoring the importance of involving end users throughout the design process (36, 37). This ensures that the app aligns with real-world workflows and addresses both technological integration challenges and behavioral factors affecting patient and clinician engagement. Additionally, the app builds on validated behavior change models, which is less commonly emphasized in comparable tools.

Opioid prescribing is a complex process that has additional considerations when reducing patients' prescriptions, and is not completely solved by the TAPR-CPM app. The goal of the CDS tool is to support clinical decision-making at the point of care, leveraging the clinical expertise of the provider and the perspective of the patient. Therefore, integrated components of the solution like creating the taper plan (i.e., calculating doses corresponding to tapering speed) only partially address the tapering discussion. The technical solution does not directly support ongoing patient fear of withdrawal or abandonment, challenges that can only be addressed by improved patient-provider communication (10). Consistent with interview findings about desired support during the process of tapering rather than identifying patients appropriate for tapering, the app does not evaluate individual patients' risk or suggests patients that should be considered for enrollment

The patient-facing app was limited in its functionality given the need to strike a careful balance in providing medication information but not medical advice. The clinical teams expressed concern about the need to modify and respond to patient-generated data in real-time which presented workflow and legal challenges. Therefore, patient facing materials included disclaimers on the intention of the TAPR-CPM app. Because of technical and protocol limitations, the app was not designed to write back directly to the EHR, but instead leveraged a data hub to collect provider generated data, primarily the opioid tapering plan. Writing into the EHR is a long-standing challenge, particularly in medication prescribing Fast Healthcare Interoperability Resources (FHIR) applications which unintentionally bypass EHR vendor medication safeguards. FHIR specifies standards for exchange of health data between technical systems in healthcare (38). Addressing this functionality was outside the scope of this project.

Our work also demonstrated the feasibility of designing a “lite” version of the app, which requires fewer technical resources and minimizes dependence on complex healthcare system interoperability. This streamlined version could be particularly suitable for deployment in lower-resource settings or healthcare systems facing significant interoperability challenges. However, while the technical feasibility of the lite version was established, it was not tested with patients or providers. Future research should explore patient and provider perspectives on the lite version to assess its effectiveness in supporting opioid tapering, identify any unique barriers or facilitators in its use, and determine whether the simplified approach can achieve comparable outcomes to the fully integrated app. Such studies will be essential to refine the lite version and expand its applicability across diverse healthcare settings.

Our goal was to design the app iteratively with significant stakeholder engagement and feedback at many timepoints, but often the feedback was conflicting or introduced privacy, policy, or legal challenges. For example, patient stakeholders requested ongoing screening through the app for depression, but the app was not designed to alert a provider to a medical emergency and would introduce both technical (alerting) and legal liability issues. Family members and caregivers of patients suggested the ability for a secondary log-in to see the patient's app input in order to support their medical and emotional needs which would introduce privacy challenges. We considered every stakeholders' feedback to guide app design and functionality but had to balance practical challenges for the TAPR-CPM app implementation.

Our research also revealed healthcare system challenges that could impact the successful adoption of the app. One notable barrier is the complexity of patient portal authentication, which may hinder access for patients unfamiliar with digital tools or those experiencing technological barriers. Additionally, ensuring consistent technical support and addressing variable digital literacy levels across patient populations are critical for widespread adoption. To address these challenges, future implementation efforts should prioritize proactive enrollment strategies, such as assisting patients with portal registration during clinical visits and offering automatic enrollment with immediate access upon sign-up. Simplifying authentication processes and providing tailored support could reduce delays, improve usability, and promote equitable engagement.

## 5 Conclusions and next steps

The TAPR-CPM app was developed through a human factors, user-centered design approach. Methodologies like stakeholder interviews with patients, caregivers, providers, and developers; an ethnographic approach for workflow analysis and process mapping; design workshops with PCPs and pain specialists; and usability testing support the design and development of to develop a user-friendly experience with highly accessible technology that met stakeholder workflow and decision-making needs. Our next steps include wider scale implementation of the apps in a large healthcare system by engaging with healthcare providers and patients with chronic noncancer pain.

## Data Availability

The qualitative datasets presented in this article (i.e., interview transcripts) are not readily available to protect the privacy of participants and comply with IRB regulations about protections for human subjects research participants. Requests to access the datasets should be directed to kristen.e.miller@medstar.net.

## References

[1] Treede R-D Rief W Barke A Aziz Q Bennett MI Benoliel R A classification of chronic pain for ICD-11. Pain. (2015) 156(6):1003–7. 10.1097/j.pain.0000000000000160 25844555 PMC4450869

[2] Rikard SM. Chronic pain among adults—United States, 2019–2021. MMWR Morb Mortal Wkly Rep. (2023) 72:379–85. 10.15585/mmwr.mm7215a1 37053114 PMC10121254

[3] Policy BoHS, Research CoAP, Care. Relieving Pain in America: A Blueprint for Transforming Prevention, Care, Education, and Research. Washington, DC: National Academies Press (2011). 22553896

[4] Dowell D Haegerich TM Chou R. CDC Guideline for prescribing opioids for chronic pain—United States, 2016. Jama. (2016) 315(15):1624–45. 10.1001/jama.2016.1464 26977696 PMC6390846

[5] Chou R Turner JA Devine EB Hansen RN Sullivan SD Blazina I The effectiveness and risks of long-term opioid therapy for chronic pain: a systematic review for a national institutes of health pathways to prevention workshop. Ann Intern Med. (2015) 162(4):276–86. 10.7326/M14-2559 25581257

[6] Schneiderhan J Clauw D Schwenk TL. Primary care of patients with chronic pain. JAMA. (2017) 317(23):2367–8. 10.1001/jama.2017.5787 28520835

[7] Harle CA Bauer SE Hoang HQ Cook RL Hurley RW Fillingim RB. Decision support for chronic pain care: how do primary care physicians decide when to prescribe opioids? A qualitative study. BMC Fam Pract. (2015) 16:1–8. 10.1186/s12875-015-0264-3 25884340 PMC4399157

[8] Frank JW Levy C Matlock DD Calcaterra SL Mueller SR Koester S Patients’ perspectives on tapering of chronic opioid therapy: a qualitative study. Pain Med. (2016) 17(10):1838–47. 10.1093/pm/pnw078 27207301 PMC6281041

[9] Penney LS Ritenbaugh C DeBar LL Elder C Deyo RA. Provider and patient perspectives on opioids and alternative treatments for managing chronic pain: a qualitative study. BMC Fam Pract. (2016) 17:1–15. 10.1186/s12875-016-0566-0 28403822 PMC5390355

[10] Matthias MS Johnson NL Shields CG Bair MJ MacKie P Huffman M I'm not gonna pull the rug out from under you”: patient-provider communication about opioid tapering. J Pain. (2017) 18(11):1365–73. 10.1016/j.jpain.2017.06.008 28690000 PMC6219456

[11] Henry SG Paterniti DA Feng B Iosif A-M Kravitz RL Weinberg G Patients’ experience with opioid tapering: a conceptual model with recommendations for clinicians. J Pain. (2019) 20(2):181–91. 10.1016/j.jpain.2018.09.001 30243859 PMC6349552

[12] Kosakowski S Benintendi A Lagisetty P Larochelle MR Bohnert AS Bazzi AR. Patient perspectives on improving patient-provider relationships and provider communication during opioid tapering. J Gen Intern Med. (2022) 37(7):1722–8. 10.1007/s11606-021-07210-9 34993861 PMC9130417

[13] Holmes MM Lewith G Newell D Field J Bishop FL. The impact of patient-reported outcome measures in clinical practice for pain: a systematic review. Qual Life Res. (2017) 26:245–57. 10.1007/s11136-016-1449-5 27815820 PMC5288411

[14] Black N. Patient reported outcome measures could help transform healthcare. Br Med J. (2013) 346:f167. 10.1136/bmj.f167 23358487

[15] Greenhalgh J Gooding K Gibbons E Dalkin S Wright J Valderas J How do patient reported outcome measures (PROMs) support clinician-patient communication and patient care? A realist synthesis. J Patient Rep Outcomes. (2018) 2:1–28. 10.1186/s41687-018-0061-6 30294712 PMC6153194

[16] Rothman ML Beltran P Cappelleri JC Lipscomb J Teschendorf B. Patient-reported outcomes: conceptual issues. Value Health. (2007) 10(2):S66–75. 10.1111/j.1524-4733.2007.00269.x 17995476

[17] Kroenke K Cheville A. Management of chronic pain in the aftermath of the opioid backlash. JAMA. (2017) 317(23):2365–6. 10.1001/jama.2017.4884 28494058

[18] O'Connor AM Rostom A Fiset V Tetroe J Entwistle V Llewellyn-Thomas H Decision aids for patients facing health treatment or screening decisions: systematic review. Br Med J. (1999) 319(7212):731–4. 10.1136/bmj.319.7212.731 10487995 PMC28223

[19] Ballantyne JC Sullivan MD. Intensity of chronic pain—the wrong metric. N Engl J Med. (2015) 373(22):2098–9. 10.1056/NEJMp1507136 26605926

[20] Littlejohn R Barrientos RR Boxley C Miller K. Owning Attention: Applying Human Factors Principles to Support Clinical Decision Support. Recent Advances in Digital System Diagnosis and Management of Healthcare: IntechOpen. (2020).

[21] Schubel L Stein L Romero R Miller K, editors. Mitigating cardiovascular risk through user informed clinical decision support. Proceedings of the International Symposium on Human Factors and Ergonomics in Health Care. Los Angeles, CA: SAGE Publications Sage CA (2020).

[22] Schubel L Muthu N Karavite D Arnold R Miller K. Design for cognitive support. Des Health. (2020):227–50. 10.1016/B978-0-12-816427-3.00012-9

[23] Xiao Y Miller K Werner N Smith K Hendrix N Hemmelgarn C editors. Co-Design with patients for improving patient safety: strategies, barriers and pitfalls. Proceedings of the Human Factors and Ergonomics Society Annual Meeting. Los Angeles, CA: SAGE Publications Sage CA (2023). 10.1177/21695067231192416 PMC10782182 38213999

[24] Zamenopoulos T Alexiou K. Co-design as collaborative research: Bristol University/AHRC Connected Communities Programme. (2018).

[25] Rotteau L Magaz M Wong BM Shearkhani S Shabani M Pradhan R Community-engaged co-design of a quality improvement capacity building program within an integrated health system in Ontario, Canada. J Integr Care. (2024) 32:303–12. 10.1108/JICA-05-2023-0028

[26] Feldstein AC Glasgow RE. A practical, robust implementation and sustainability model (PRISM) for integrating research findings into practice. Jt Comm J Qual Patient Saf. (2008) 34(4):228–43. 10.1016/S1553-7250(08)34030-6 18468362

[27] Pocket guide: tapering opioids for chronic pain. Centers for Disease Control and Prevention (U.S.). (2016).

[28] Berna C Kulich RJ Rathmell JP. Tapering long-term opioid therapy in chronic noncancer pain: evidence and recommendations for everyday practice. Mayo Clin Proc. (2015) 90(6):828–42. 10.1016/j.mayocp.2015.04.003 26046416

[29] Card P. VA/DoD Clinical Practice Guideline for Opioid Therapy for Chronic Pain. Washington, DC: Department Veterans Aff Department Defense (2017). p. 3.

[30] Darnall BD Ziadni MS Stieg RL Mackey IG Kao MC Flood P. Patient-Centered prescription opioid tapering in community outpatients with chronic pain. JAMA Intern Med. (2018) 178(5):707–8. 10.1001/jamainternmed.2017.8709 29459978 PMC5876887

[31] Dowell D Compton WM Giroir BP. Patient-centered reduction or discontinuation of long-term opioid analgesics: the HHS guide for clinicians. JAMA. (2019) 322(19):1855–6. 10.1001/jama.2019.16409 31600366 PMC7145754

[32] Strauss A Corbin J. Grounded theory methodology: An overview. (1994).

[33] Tschimmel K. editor Design Thinking as an effective Toolkit for Innovation. ISPIM Conference Proceedings; 2012: The International Society for Professional Innovation Management (ISPIM).

[34] Greenes RA Bates DW Kawamoto K Middleton B Osheroff J Shahar Y. Clinical decision support models and frameworks: seeking to address research issues underlying implementation successes and failures. J Biomed Inform. (2018) 78:134–43. 10.1016/j.jbi.2017.12.005 29246790

[35] Marcial LH Jacobs S Goode SA Booth G Barnes K Renaud J Clinical Decision Support for Chronic Pain Management. Rockville, MD: Agency for Healthcare Research and Quality (2024). Contract No.: 24-0074.

[36] Devan H Perry MA Yaghoubi M Hale L. A coalition of the willing": experiences of co-designing an online pain management programme (iSelf-help) for people with persistent pain. Res Involv Engagem. (2021) 7(1):28. 10.1186/s40900-021-00275-0 33975653 PMC8112221

[37] Elbers S van Gessel C Renes RJ van der Lugt R Wittink H Hermsen S. Innovation in pain rehabilitation using co-design methods during the development of a relapse prevention intervention: case study. J Med Internet Res. (2021) 23(1):e18462. 10.2196/18462 33470937 PMC7857944

[38] What is FHIR? Available online at: https://www.healthit.gov/sites/default/files/2019-08/ONCFHIRFSWhatIsFHIR.pdf (Accessed February 01, 2025).

